# LINC00491 promotes cell growth and metastasis through miR-324-5p/ROCK1 in liver cancer

**DOI:** 10.1186/s12967-021-03139-z

**Published:** 2021-12-07

**Authors:** Wei Wang, Tao Yang, Dongsheng Li, Yinpeng Huang, Guang Bai, Qing Li

**Affiliations:** 1 Department of General Surgery, The First Affiliated Hospital of Jinzhou Medical University, Jinzhou, 121000 China; 2 Department of Nephrology, The Third Affiliated Hospital of Jinzhou Medical University, No. 2 Section 5 Heping Road, Jinzhou, 121000 China

**Keywords:** LINC00491, Liver cancer, miR-324-5p, ROCK1, Metastasis, lncRNA

## Abstract

**Background:**

LINC00491 was involved in some tumors development, but its function in liver cancer has not been reported. This study aimed to investigate LINC00491 expression and function in liver cancer progression.

**Methods:**

Sixty liver cancer cases were enrolled. LINC00491, miR-324-5p and rho-associated kinase 1 (ROCK1) expression in liver cancer patients and cells were detected by quantitative reverse transcription-polymerase chain reaction and Western blot. HUH-7 and SK-Hep-1 cells were transfected to modulate LINC00491, miR-324-5p and ROCK1 expression. Cell counting kit-8 assay, colony formation assay, wound healing assay, Transwell experiment, Tunel assay and flow cytometry were performed to detected HUH-7 and SK-Hep-1 cells proliferation, migration, invasion, apoptosis and cell cycle. Biotin-RNA pull-down assay and Dual-Luciferase Reporter Assay was performed to detect the binding among LINC00491, miR-324-5p and ROCK1. Xenograft tumor and lung metastasis was performed using nude mice. Xenograft tumor and lung tissues of mice were experienced immunohistochemistry and hematoxylin–eosin staining.

**Results:**

LINC00491 was highly expressed in liver cancer cases, associating with poor prognosis. si-LINC00491 inhibited proliferation, colony formation, invasion, migration, and induced cell cycle G1 arrest and apoptosis in HUH-7 and SK-Hep-1 cells. LINC00491 overexpression showed opposite effects. LINC00491 promoted ROCK1 expression by reducing miR-324-5p. miR-324-5p up-regulation or ROCK1 knockdown reversed LINC00491 promotion on liver SK-Hep-1 cells malignant phenotype. LINC00491 facilitated xenograft tumor growth and lung metastasis in mice.

**Conclusion:**

LINC00491 was highly expressed in liver cancer patients, associating with poor prognosis. LINC00491 facilitated liver cancer progression by sponging miR-324-5p/ROCK1. LINC00491 might be a potential treatment target of liver cancer.

## Introduction

Globally, liver cancer is one of the most common malignant tumors. Epidemiological studies have shown that there are significant geographical differences in the incidence of liver cancer; Asia, especially China, has a higher incidence [1]. Liver cirrhosis caused by a chronic hepatitis virus infection, including hepatitis B virus (HBV) and hepatitis C virus (HCV), is the most common factor for the occurrence of liver cancer [2]. China is a country with a large population and a high incidence of hepatitis. Chronic hepatitis virus infection caused by liver fibrosis and cirrhosis are the most important risk factors for liver cancer in China [3]. The number of new liver cancer patients in China accounts for approximately 55%, and the deaths account for approximately 45% of the world statistics [4]. The early clinical symptoms of liver cancer are not obvious, and thus, are often neglected. It is usually diagnosed in middle or advanced stages, where there is a high degree of malignancy, rapid progression, easy recurrence of metastasis after treatment, and poor prognosis [5]. Therefore, although the treatment for liver cancer has made great progress through radical surgery, targeted drugs, and interventional therapy which has benefited many patients, the mortality rate of liver cancer remains high [6].

The tumorigenesis and progression of liver cancer are complex biological processes involving the dysfunction of a variety of cancer-related molecules and genes. In the last few decades, research on the molecular mechanisms for the diagnosis and treatment of liver cancer has made great progress, but the key factors underlying pathogenesis remain unclear [7]. In recent years, increasing evidence shows that the non-coding RNAs, such as long noncoding RNAs (lncRNAs) and microRNAs (miRNAs), participate and play a crucial role in several biological processes at multiple levels [8]. Existing study shows that many lncRNAs and miRNAs play a role in the suppression or promotion of tumor function in several cancers, and can be used as diagnostic markers for predicting the risk of tumorigenesis [9]. For example, lncRNA nuclear factor-kappa B interacting long non-coding RNA (NKILA) can inhibit metastasis in adenocarcinoma by blocking inhibitor of nuclear factor-kappa B (IκB) acidification. LncRNA HOX transcript antisense RNA (HOTAIR) can promote the metastasis of breast cancer by inducing chromatin rearrangement [10]. LncRNA metastasis-associated lung adenocarcinoma transcript 1 (MALAT-1) plays an important regulatory role in metastasis of lung cancer cells [11], and NKILA can inhibit metastasis of pancreatic cancer by blocking IκB phosphorylation [12]. To date, several lncRNAs are closely related to the occurrence and development of liver cancer, such as HOTAIR [13], hepatocellular carcinoma up-regulated EZH2-associated long non-coding RNA (HEIH) [14], down-regulated expression by HBx (Dreh) [15], maternally expressed gene 3 (MEG3) [16] etc. The discovery of the biological functions of lncRNAs provides new dawn for investigating the molecular mechanisms and further exploration of new treatment options for tumors.

LINC00491 is located on human chromosome 5q21.1. Several bioinformatic analyses based on endogenous RNA Networks show that LINC00491 is positively correlated with the overall survival in breast cancer [17] and colorectal cancer [18] patients. It is an independent prognostic biomarker for endometrial cancer [19]. However, to the best of our knowledge, the biological functions of LINC00491 in liver cancer have not been reported. This study aimed to investigate the expression and biological functions of LINC00491 in liver cancer. Through bioinformatics analysis, miR-324-5p was shown to possess binding sites of LINC00491 and rho-associated kinase 1 (ROCK1). Thus, this paper researched the intrinsic molecular mechanism of LINC00491 in regulating liver cancer progression with miR-324-5p/ROCK1 as the axis. It was hoped that LINC00491 could be identified as an effective target for liver cancer treatment.

## Method and materials

### The Cancer Genome Atlas (TCGA) and the Gene Expression Omnibus (GEO) analysis

LINC00491 expression data in non-tumor tissues and tumor tissues of liver cancer cases was downloaded and analyzed from TCGA and GEO (GSE55191 and GSE58043) database.

### Clinical tissue samples

All clinical specimens (60 cases) were derived from patients who underwent surgical treatment for liver cancer in the Third Affiliated Hospital of Jinzhou Medical University between 2011 and 2013. The selected liver cancer tissues were confirmed by pathological analysis, and the para-cancerous liver tissues obtained were at least 3 cm away from the tumor. Specimens were stored in liquid nitrogen immediately after excision. The clinical data of patients were shown in Table 1. This study design was approved by the ethics committee of the Third Affiliated Hospital of Jinzhou Medical University in line with the Declaration of Helsinki.

**Table 1:** Relationship of LINC00491 expression and the clinicopathologic features of the liver cancer patients

Characteristics	Number of patients	LINC00491 Low expression (< median)	LINC00491 High expression (≥ median)	P value
Number	60	28	32	
Ages (years)				0.398
< 65	30	13	17	
≥ 65	30	15	15	
Gender				0.058
Female	29	10	19	
Male	31	18	13	
Tumor size (cm)				0.053
≤ 3	33	19	14	
> 3	27	9	18	
Tumor stage				0.014*
I	22	15	7	
II	17	8	9	
III/IV	21	5	16	
Lymph node metastasis				0.010*
Positive	32	10	22	
Negative	28	18	10	

*P < 0.05

### Cell lines and transfection

The normal human liver L02 and liver cancer HUH-7, HepG2, HUH-6 and SK-Hep-1 cell lines (China Center for Type Culture Collection) were cultured in Dulbecco’s modified eagle medium (DMEM) supplemented with 10% fetal bovine serum (FBS) at 37 °C, 5% CO_2_. The pcDNA3.1-LINC00491 vectors, empty vectors, si-LINC00491 and corresponding negative control (siNC), si-ROCK1 miR-324-5p mimics were designed and synthesized by GenePharma Co., Ltd. (Shanghai, China). HUH-7 and SK-Hep-1 cell lines were subjected to transfection. The transfection was performed with Lipofectamine™ 2000 reagent (Invitrogen) according to the manufacturer's instructions.

### Subcellular localization assay

Fluorescence in situ hybridization (FISH) was used to detect the localization of LINC00491 in HUH-7 cells. The lncRNA FISH Probe Mix (RIBOBIO) was used. The HUH-7 cells were trypsinized and re-suspended in fresh media to a final concentration of 5 × 10^4^ cells/ml. Then 400 μl/well of the cell suspension was added to a 24-well plate and incubated for 24 h. Then the cells were washed with PBS and fixed with 4% paraformaldehyde. Subsequently, the cells were blocked with 0.5% Triton X-100, incubated with prehybridization solution, and hybridized with LINC00491 probe overnight at 37 °C. The nuclei were stained with 4', 6-diamidino-2-phenylindole (DAPI) and the cells were observed under a confocal laser microscope (Leica, Mannheim, Germany).

### Cell proliferation assay

After transfection of HUH-7 and SK-Hep1 cells with the appropriate plasmids, the cells were trypsinized and re-suspended in fresh media with a final concentration of 1 × 10^5^ cells/ml. Then 100 μl/well of the cell suspension was added to a 96-well plate. There were six sub-wells for each group. The cells were cultured at 37 °C, 5% CO_2_ for 24, 48, and 72 h, respectively. After that, 10 μl of cell counting kit-8 (CCK-8) reagent was added to each well and incubated for 2 h. The absorbance of each well was measured at 450 nm using a multi-well microplate reader (Bio-TekR Instruments, Inc., Winooski, Vermont, USA).

### Terminal transferase-mediated DNA end labelling (TUNEL) assay

After the transfection of HUH-7 and SK-Hep1 cells with appropriate plasmids, the cells were trypsinized and re-suspended in fresh media to a final concentration of 1 × 10^5^ cells/ml. Thereafter, 400 μl/well of the cell suspension was added to a 24-well plate and cultured for 24 h. The cells were washed with phosphate buffered saline (PBS) and fixed with 4% paraformaldehyde. Cells were permeabilized with 0.1% Triton X100 in PBS for 2 min on ice. After washing with PBS, the cells were treated with 50 μl TUNEL test solution for 60 min at 37 ℃ in dark. The cells were then sealed with anti-fluorescence quenching liquid and observed under a fluorescence microscope (EX: 450–500 nm, EM: 515–565 nm).

### Flow cytometry

After transfecting the plasmids into HUH-7 and SK-Hep1 cells, the cells were trypsinized and re-suspended in fresh media to a final concentration of 1 × 10^5^ cells/ml. After wards, 1 ml/well of the cell suspension was added to a 6-well plate and cultured for 24 h. Subsequently, the cells were collected after trypsinization and washed with ice-cold PBS. The supernatant was removed after centrifugation. The cells were re-suspended in 300 μl of PBS containing 5% bovine serum albumin (BSA) and fixed with 700 μl of ethanol at 4 °C for 24 h. The cells were washed with PBS and centrifuged to remove the ethanol. The cells were re-suspended in 100 μl of PBS containing 1 μl of ribonuclease A (RNase A) (10 mg/μl) and incubated at 37 °C for 30 min. Then, cells were stained with 50 μl propidium iodide (PI) for 30 min. Cell cycle distribution was analyzed using a flow cytometry on the FACSCalibur system (Becton Dickinson, San Jose, CA, USA).

### Colony formation assay

After transfecting HUH-7 and SK-Hep1 cells with the appropriate plasmids, they were trypsinized and re-suspended in fresh media to a final concentration of 1 × 10^4^ cells/ml. Then 100 μl/well of cell suspension was added to a 6-well plate containing 1 ml of 30% FBS supplemented in DMEM. The cells were cultured for 14 days and culture medium was changed every 3 days. The supernatant was discarded, and the cells were washed twice with ice-cold PBS. Then, 500 μl of 95% ethanol was added to each well for 10 min. After ethanol being discarded, the cells were washed twice with PBS. Cells were stained with 500 μl of 0.5% crystal violet for 10 min. The crystal violet was discarded, and the cells were washed twice with PBS. At last, the cells were observed under a microscope (Nikon, Tokyo, Japan) to count the colony formation (no less than 50 cells) number.

### Wound healing assay

After transfecting HUH-7 and SK-Hep1 cells with the appropriate plasmids, they were trypsinized and re-suspended in fresh media to a final concentration of 1 × 10^6^ cells/ml. Then 1 ml/well of the cell suspension was added to a 6 mm Petri dish containing 4 ml of serum-free DMEM. After culturing for 12 h, a scratch was made with a pipette tip. The width of scratch gaps was recorded. The cells were cultured for 48 h. Then the width of wound gaps were recorded again and photographed.

### Cell invasion assay

Matrigel was diluted to a final concentration of 5 μg/μl and added to the upper chamber at 50 μl/well for polymerizing into a gel. After transfecting HUH-7 and SK-Hep1 cells with the appropriate plasmids, they were trypsinized and re-suspended in serum-free DMEM to a final concentration of 5 × 10^4^ cells/ml. Then 200 μl/well of the cell suspension was added to the upper chamber, and 500 μl of DMEM supplemented with 10% FBS was added to the lower chamber. After culturing for 24 h, the trans-well was taken out. The cells were washed twice with PBS and fixed with 95% alcohol. Then, 500 μl of 0.1% crystal violet was added for 30 min. The crystal violet was discarded, and the cells were washed twice with PBS. The cells on the upper surface were wiped with a cotton ball. Cells attached on the lower surface was observed under a microscope (Nikon, Tokyo, Japan) and photographed.

### Biotin-RNA pull-down assay

Through Starbase online bioinformatics analysis, the potential target miRNAs for LINC00491 were predicted. According to the degree of target binding and the influence of these miRNAs in liver cancer, miR-324-5p was finally selected. Then RNA pull-down assay was applied to detect the binding between LINC00491 and miR-324-5p. The total RNA extracted from HUH-7 cells was transcribed using Biotin RNA Labeling Mix (Roche) and T7 polymerase (Promega). The biotin-RNA product was then obtained, followed by being purified using DNase I (Promega) and Rneasy Mini Kit (QIAGEN). Next, the biotin-RNA (3 μg) was heated at 90 °C for 2 min and then transferred to the ice for 2 min. RNA structure buffer (10 mM Tris pH 7.0, 0.1 M KCl, 10 mM MgCl_2_) was added and the reaction was allowed to stand for 20 min. Then, the HUH-7 cells (1 × 10^7^ cells) were suspended in 2 ml of PBS, and 2 ml of radio-immunoprecipitation assay (RIPA) buffer was added. The cells were lysed for 1 h at 4 °C. The lysate was centrifuged at 12,000*g* for 10 min at 4 °C, and the supernatant was collected in a fresh RNase-free tube. Then 1 μg biotin-labeled RNA and 500 μl radioinmunoprecipitacion (RIP) buffer were added to the tube and incubated for 1 h. Afterwards, 20 μl of washed streptavidin agarose beads were added to each tube and incubated for 30 min. The agarose beads were washed with Handee spin column. The washing solutions were collected for subsequent analysis.

### Dual-Luciferase Reporter Assay

Through Starbase online bioinformatics analysis, the potential target of miR-324-5p was predicted. According to the degree of target binding, ROCK1 was selected as the subject. Dual-luciferase reporter assay was then conducted to research the binding of ROCK1 and miR-324-5p or LINC00491. To construct the luciferase reporter vectors, ROCK1 wild type (wt) and mutant type (mut) sequence was amplified by polymerase chain reaction (PCR) and inserted into the pmirGLO vector (Promega, Madison, WI, USA) (pmirGLO-ROCK1-wt, pmirGLO-ROCK1-mut). HEK-293 T cells were seeded into 24-well plates and cultured for 24 h. The pmirGLO vectors were co-transfected with miR-324-5p mimics, mimics NC, pcDNA3.1-LINC00491 vectors, empty vectors, si-LINC00491 or siNC into HEK-293 T cells. After 48 h of transfection, the media was discarded. The cells were washed with ice-cold PBS. The luciferase activity was detected using a Dual-Luciferase® Reporter Assay System (Promega, Madison, WI, USA).

### Quantitative reverse transcription-polymerase chain reaction (qRT-PCR)

Total RNA of the tissues or cells was extracted using the TRIzol reagent. First, the mRNA was reverse transcribed to cDNA using PrimeScript™ RT reagent kit with gDNA Eraser (TAKARA). The reaction system consisted of the following: total RNA 1 μg, gDNA Eraser 1 μl, 5 × g DNA Eraser buffer 2 μl, PrimeScrtipt RT Enzyme Mix 1 μl, 5 × PrimeScrtipt Buffer 4 μl, RT Primer Mix 4 μl. RNAse free water was added to a total volume of 20 μl. Reaction conditions were as follows: 37 °C for 15 min, 85 °C for 5 s. The cDNA product was immediately used as a template for the PCR reaction. Glyceraldehyde-3-phosphate dehydrogenase (GAPDH) was used as an internal control. miRNA was reverse transcribed to cDNA was performed using the miRNA First Strand cDNA Synthesis kit (Sangon Biotech). The reaction system consisted of the following: 2 × miRNA RT Solution mix 10 μl, miRNA RT Enzyme mix 2 μl, total RNA 2 μg. RNAse free water was added to a total volume of 20 μl. Reaction conditions were as follows: 37 °C for 60 min, 85 °C for 5 min. The cDNA product was immediately used as the template for the PCR reaction. U6 was used as an internal control.

Subsequently, the cDNAs were used for a qPCR assay with the SYBR Premix Ex Taq™ kit. The reaction system consisted of the following: 2 × SYBR Premix Ex Taq™ 12.5 μl, forward and reverse primers each 0.5 μl, cDNA sample 2 μl. dH_2_O was added to a total volume of 25 μl. Reaction conditions were as follows: 94 °C for 30 s, 45 cycles of (95 °C for 5 s, 60 °C for 30 s, 72 °C for 30 s). GAPDH was used as internal control for ROCK1 and LINC00491, while U6 was selected as internal control for miR-324-5p. The relative expression levels were calculated by the 2^−ΔΔCT^ method. The primers synthesized by Sangon Biotech (Shanghai, China) were as follows: miR-324-5p (5′-CGCATCCCCTAGGGCATTGGTG-3′), ROCK1 (F: 5′-CAAACGATATGGCTGGAAG-3′; R: 5′-TGGATTGGATTGCTCCTTA-3′), LINC00491 (F: 5′-ATGGGGTGGTGGTATTTTC-3′; R: 5′-CCTGTTTGGGTCTTCTTCA-3′). GAPDH (F: 5′-CCTTCCGTGTCCCCACT-3′; R: 5′-GCCTGCTTCACCACCTTC-3′).U6 (F: 5′-AACGCTTCACGAATTTGCGT-3′; R: 5′-CTCGCTTCGGCAGCACA-3′).

### Western blotting

Tissue samples were added to a mixture of pre-chilled RIPA lysate and protease inhibitor cocktail (1:50), and then homogenized by grinding on ice. The mixture was centrifuged at 12,000 r/min for 10 min at 4 °C. The supernatant was collected. After the cells were harvested, a mixture of pre-chilled RIPA lysate and protease inhibitor (1: 50) was added, mixed for 5 min at 4 °C, centrifuged at 12,000 r/min for 10 min at 4 °C. The supernatant was collected. The protein concentration was determined using a BCA protein assay kit (Beyotime, Shanghai, China). Then the 5 × loading buffer was added into each protein sample, followed by being boiled for 10 min in water bath. The protein samples were subjected to sodium dodecyl sulfate–polyacrylamide gel electrophoresis (SDS-PAGE) electrophoresis. After separation, a "transfer sandwich" was prepared for wet polyvinylidene fluoride (PVDF) membrane transfer. The membrane was washed in Tris-buffered saline with 0.5% Tween 20 (TBST) for 5 min. Then the membrane was blocked with 5% skim milk for 2 h on a shaker at room temperature. The membrane was incubated with the primary antibodies overnight at 4 °C, including anti-ROCK1 (1:1000, ab134181, Abcam, Shanghai, China) and anti-β-actin (1:1000, ab8226, Abcam, Shanghai, China). The membrane was washed four times with TBST and then incubated with horseradish peroxidase (HRP) labeled goat anti-mouse secondary antibody (1:2000, ab6789, Abcam, Shanghai, China) at 37 °C for 1.5 h. The immunoreactive protein bands were visualized by enhanced chemiluminescence (ECL) on an Amersham prime ECL Plus detection system (GE Healthcare Life Sciences, Buckinghamshire, United Kingdom).

### Xenografting subcutaneous tumor and lung metastasis

The SPF Balb/c (nu/nu) male nude mice (n = 24, 4 weeks, 20–24 g) were purchased from Shanghai Bangyao Biotech (Shanghai, China). Animal research has been approved by the animal ethics committee. The mice were housed in SPF-level sterile rooms (24 ± 2 °C) and given free access to feed and water.

LINC00491 over-expression vectors or NC were successfully transfected into SK-Hep-1 cells. The transfected SK-Hep-1 cells were trypsinized and re-suspended in PBS to a final concentration of 1 × 10^7^ cells/ml. The cells (1 ml/mouse) were injected into the left side of the groin of nude mice (n = 6 per group). The long diameter (a) and short diameter (b) of tumor tissue was measured using a vernier caliper and calculated by (a × b^2^)/2. After 3 weeks, the nude mice were euthanized, the tumor tissues were collected, weighed, and stored at − 80 °C.

Additionally, lung metastasis experiment was performed by injection the transfected SK-Hep-1 cells (1 × 10^7^ cells per mouse) via tail vein (6 mice per group). Mice were then housed for 6 weeks with free access to food and water. After 30 days, mice were euthanized. The whole lung tissues were obtained and stored at −80 °C.

### Hematoxylin–eosin(H&E) and immunohistochemistry (IHC) staining

The xenograft tumor tissues and lung tissues of mice were fixed with 10% paraformaldehyde, embedded in paraffin, and then cut into sections of 4 μm thickness. The sections were dewaxed using xylene and rehydrated using successive gradient concentrations of ethanol. For H&E staining, the lung tissue sections were first stained with hematoxylin and then with eosin. Then the sections were dehydrated and sealed. For IHC analysis, xenograft tumor tissue sections were treated with 3% H_2_O_2_ for 10 min at room temperature to inactivate the endogenous enzyme. The sections were immersed in a 0.01 M citrate buffer (pH = 6.0) and heated to boiling in a microwave oven. After cooling, samples were blocked with 5% BSA blocking solution at room temperature for 20 min. Then, the sections were incubated with Ki-67 (1:100, ab156956, Abcam, Shanghai, China) and matrix metalloproteinase-9 (MMP-9) (1:100, ab58803, Abcam, Shanghai, China) antibodies overnight at 4 °C. Subsequently, the sections were incubated with goat anti-rabbit IgG antibody (1:200, ab6721, Abcam, Shanghai, China) for 20 min at 25 °C. Diaminobenzidine (DAB) staining and hematoxylin counterstaining were performed. Sections were dehydrated and sealed in neutral resin. Images were captured using a light microscope (Olympus BX40F, Olympus, Tokyo, Japan).

### Statistical analysis

All data were presented as mean ± standard deviation (SD). The comparison between two groups was performed by two-tailed paired Student's t-test, while the comparison in multiple groups (at least three groups) was conducted by one-way analysis of variance (ANOVA). GraphPad Prism 7.0 (GraphPad Software Inc., San Diego, CA, USA) was applied for the production of statistical graphs. The survival curve was plotted by the Kaplan–Meier method and compared by log-rank tests. *P* values < 0.05 were considered statistically significant.

## Results

### LINC00491 was up-regulated in liver cancer patients and cells

**Fig. 1:**
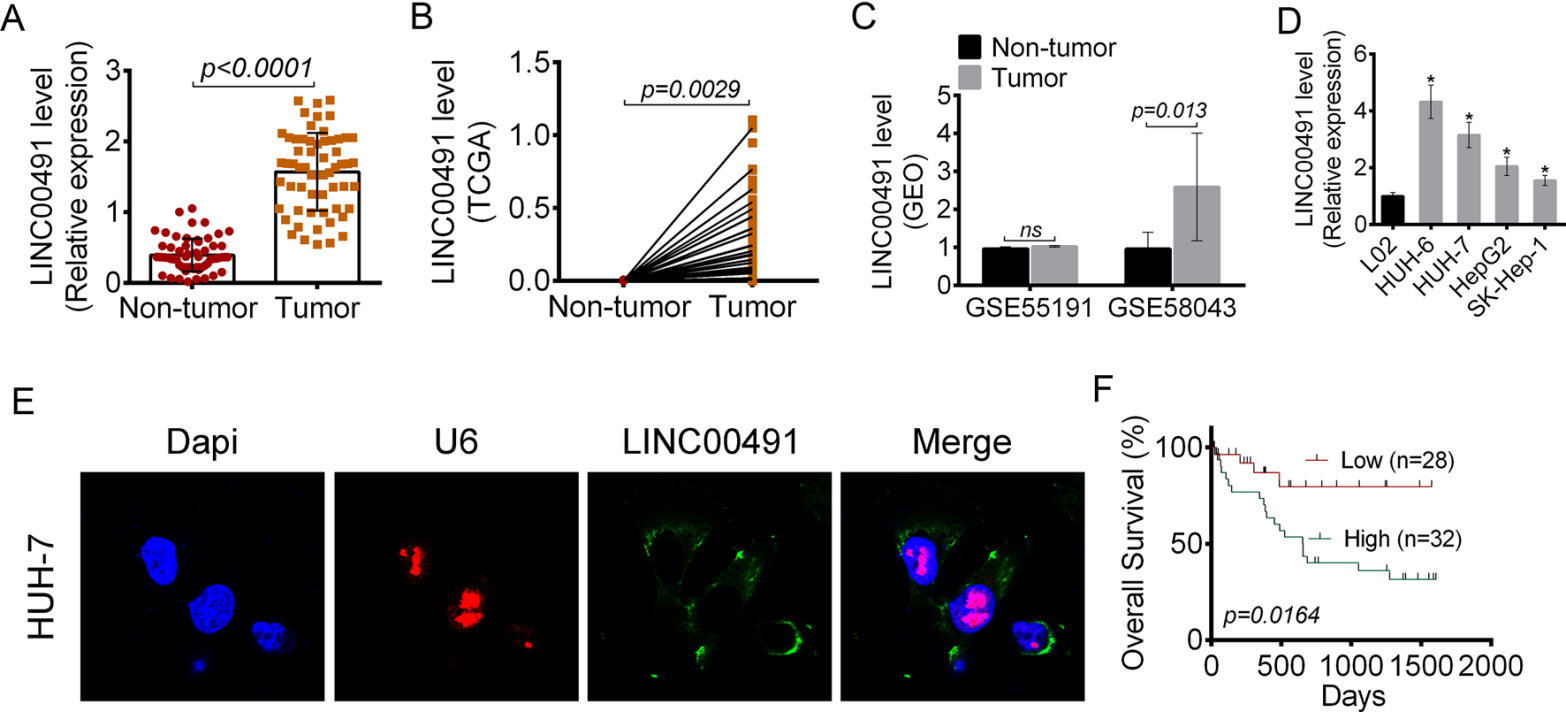
LINC00491 expression and association with prognosis in liver cancer patients. **A** The expression of LINC00491 in 60 cases of liver cancer and adjacent tissue samples. **B** The expression of LINC00491 in liver cancer tissues extracted from TCGA. **C** The expression of LINC00491 in liver cancer tissues extracted from GEO. **D** The expression of LINC00491 in liver cancer cells (HUH-6, HUH-7, HepG2 and SK-Hep-1) and normal liver cells (L02). **E** The subcellular localization of LINC00491 in HUH-7 cells. **F** Association of overall survival in liver cancer patients with high (n = 28) and low (n = 32) LINC00491 expression. *P < 0.05

The expression of LINC00491 in 60 pairs of liver cancer tissues and the corresponding adjacent normal tissues was determined by qRT-PCR. As shown in Fig. 1A, the relative expression of LINC00491 in the tumor tissues was significantly higher as compared to the normal tissues (*P* < 0.0001). TCGA and GEO databases showed that LINC00491 was highly expressed in tumor tissues than that in normal tissues (*P* = 0.0029 or 0.013) (Fig. 1B, C). Additionally, LINC00491 expression was lower in L02 cells than that in HUH-6, HUH-7, HepG2 and SK-Hep-1 cells (*P* < 0.05) (Fig. 1D). To evaluate the role of LINC00491 in liver cancer, in subsequent experiments, HUH-7 and SK-Hep-1 cells were chosen for transfection of LINC00491 over-expression vector and si-LINC00491. This study examined the subcellular localization of LINC00491 in liver cancer cells. As shown in Fig. 1E, LINC00491 was predominantly localized in the cytoplasm. Furthermore, the patients were divided into two groups based on LINC00491 expression levels. Survival curves were analyzed by the Kaplan–Meier method. As shown in Fig. 1F, relative to patients with low LINC00491 expression (< median, n = 28), patients with high LINC00491 expression (> median, n = 32) had obviously worse survival (*P* = 0.0164). The relationship between LINC00491 expression and clinicopathological characteristics of the patients was also analyzed. As shown in Table 1, high LINC00491 expression was significantly correlated with worse tumor TNM stage (*P* = 0.014) and positive lymph node metastasis (*P* = 0.010).

### Effect of dynamic expression of LINC00491 on proliferation, cell cycle and apoptosis in liver cancer cells

**Fig. 2:**
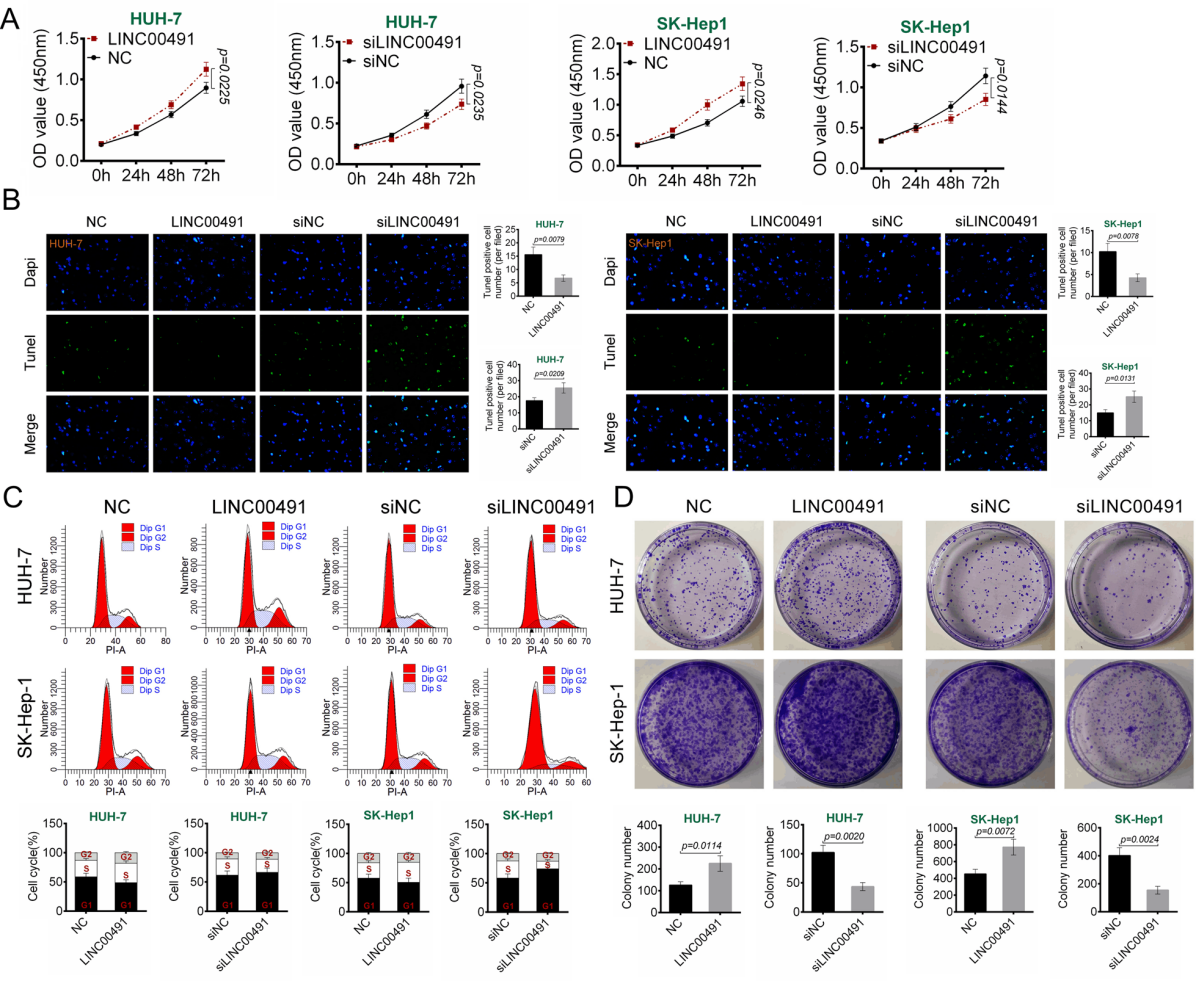
Effect of LINC00491 dynamic expression on proliferation, cell cycle and apoptosis of liver cancer cells. **A** The effect of LINC00491 over-expression vector (LINC00491) and siLINC00491 on the proliferation in HUH-7 and SK-Hep-1 cells as determined by CCK-8 assay. **B**–**D** The effect of LINC00491 over-expression vector (LINC00491) and siLINC00491 on the apoptosis (**B**), cell cycle (**C**) and colony formation (**D**) in HUH-7 and SK-Hep-1 cells

This study examined the effects of LINC00491 expression on the proliferation of HUH-7 and SK-Hep1 cells by the CCK8 assay. As shown in Fig. 2A, overexpression of LINC00491 promoted the proliferation of HUH-7 and SK-Hep1 cells, whereas the proliferation was inhibited after LINC00491 knockdown (*P* = 0.0225, *P* = 0.0235, *P* = 0.0246, or *P* = 0.0144). The effect of LINC00491 expression on apoptosis and cell cycle progression in HUH-7 and SK-Hep1 cells was further evaluated. As shown in Fig. 2B, overexpression of LINC00491 reduced the TUNEL-positive cells, whereas LINC00491 knockdown increased the TUNEL-positive cells (*P* = 0.0079, *P* = 0.0209, *P* = 0.0078, or *P* = 0.0131). Furthermore, over-expression of LINC00491 promoted cell cycle progression, whereas knockdown of LINC00491 blocked the cell cycle at the G1 phase (Fig. 2C). The effect of LINC00491 levels on colony formation was evaluated. As shown in Fig. 2D, overexpression of LINC00491 promoted colony formation in HUH-7 and SK-Hep1 cells (*P* = 0.0114 or *P* = 0.0072). However, knockdown of LINC00491 inhibited HUH-7 and SK-Hep1 cells colony formation ability (*P* = 0.0020 or *P* = 0.0024).

### Effect of dynamic expression of LINC00491 on migration and invasion in liver cancer cells

**Fig. 3:**
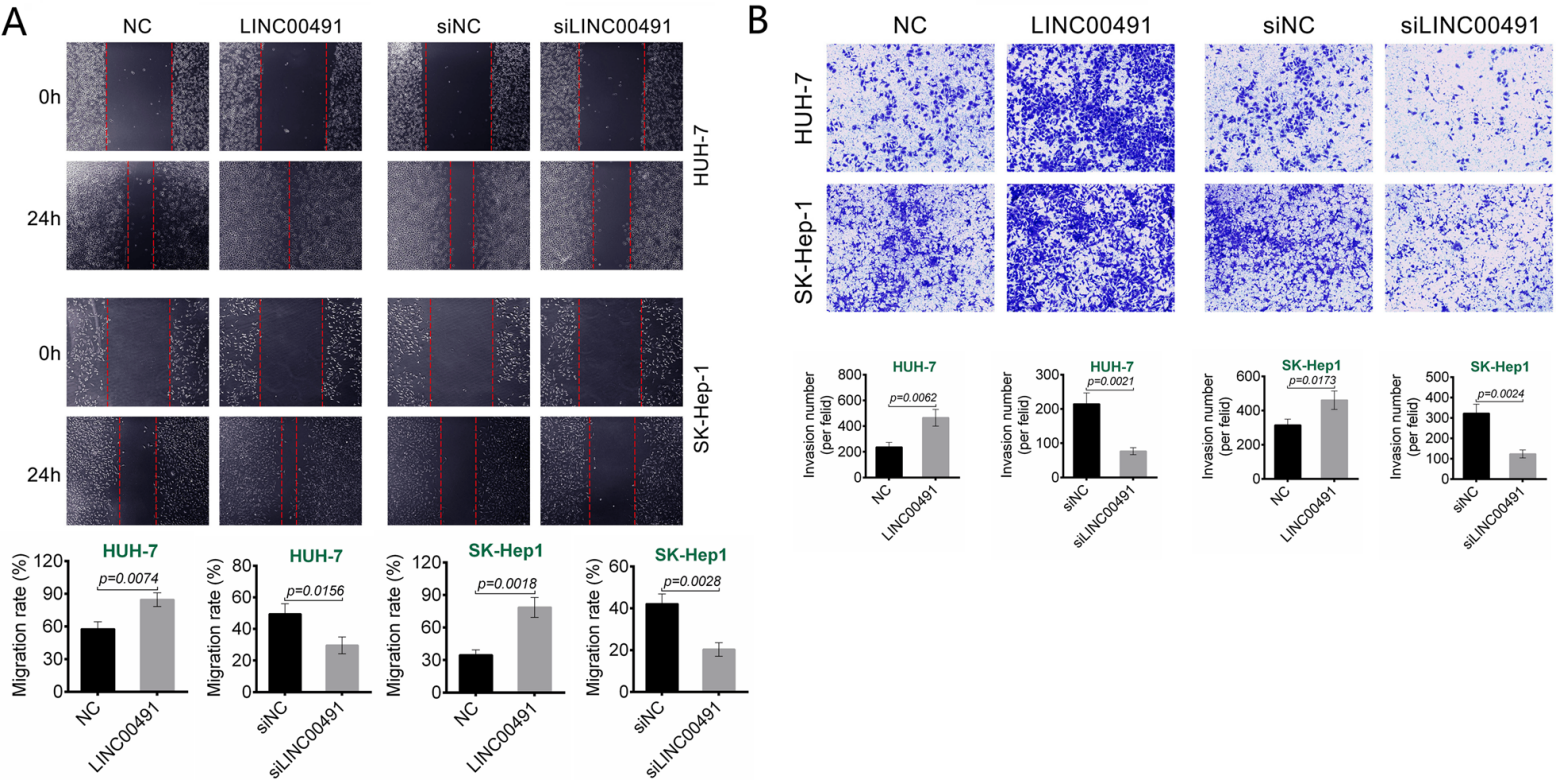
Effect of LINC00491 dynamic expression on migration and invasion of liver cancer cells. **A** The effect of LINC00491 over-expression vector (LINC00491) and siLINC00491 on the migration in HUH-7 and SK-Hep-1 cells as determined by Wound healing assay. **B** The effect of LINC00491 over-expression vector (LINC00491) and siLINC00491 on the invasion in HUH-7 and SK-Hep-1 cells as researched by Transwell experiment

Wound healing assay results were shown in Fig. 3A. Over-expression of LINC00491 promoted wound healing in HUH-7 and SK-Hep1cells as the gaps were reduced (*P* = 0.0074 or *P* = 0.0018). Oppositely, LINC00491 knockdown reduced wound healing in the two cell lines (*P* = 0.0156 or *P* = 0.0028). Transwell experiment exhibited that, over-expression of LINC00491 increased the number of invasion HUH-7 and SK-Hep1 cells (*P* = 0.0062 or *P* = 0.0173). Conversely, LINC00491 knockdown inhibited the number of invasion HUH-7 and SK-Hep1 cells (*P* = 0.0021 or *P* = 0.0024) (Fig. 3B). Thus, over-expression of LINC00491 promoted HUH-7 and SK-Hep1 cells migration and invasion.

### miR-324-5p was a target of LINC00491

**Fig. 4:**
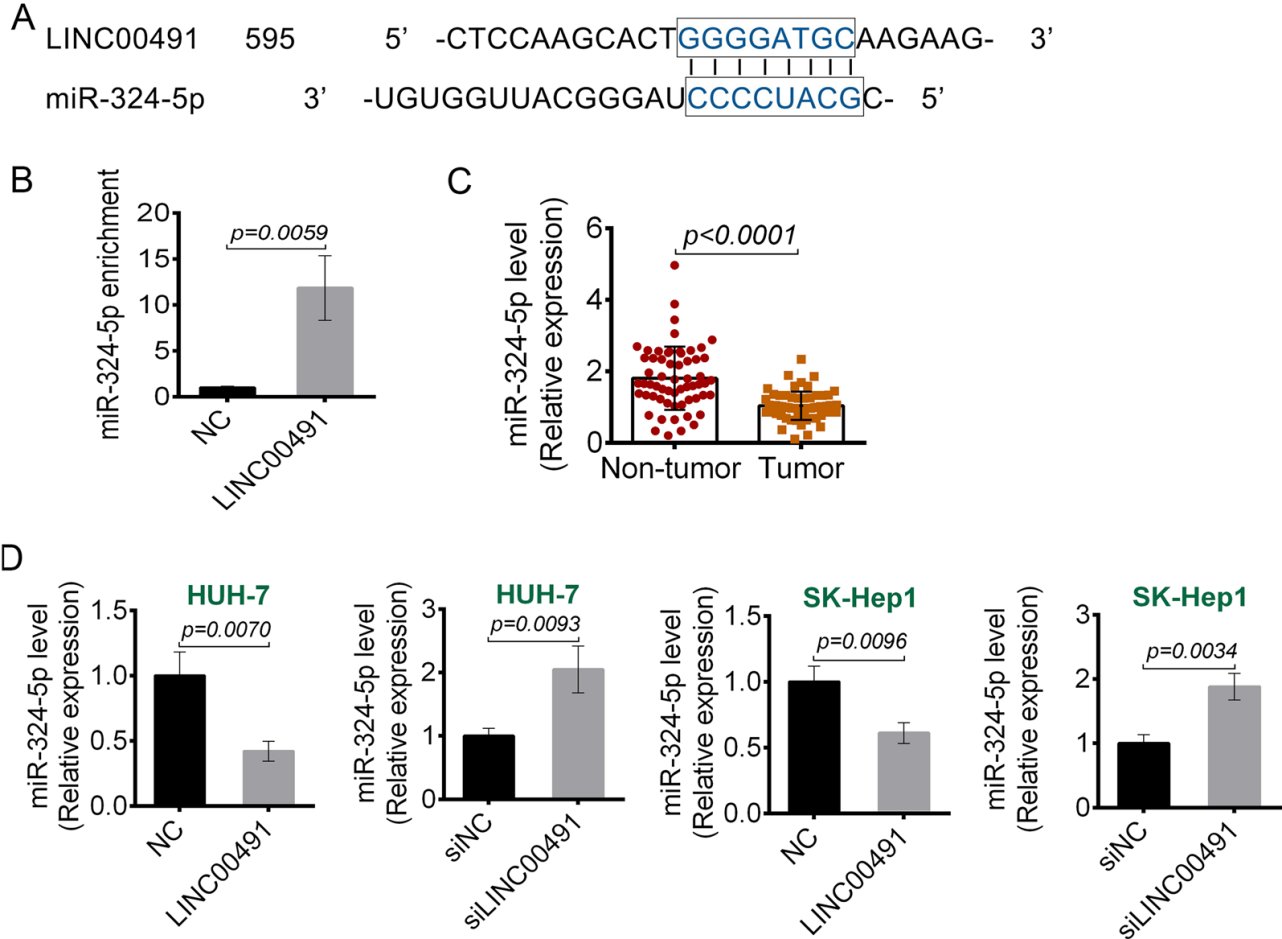
miR-324-5p was a target of LINC00491. **A** The targeting regions of LINC00491 for miR-324-5p. **B** The binding of LINC00491 and miR-324-5p was detected by Biotin-RNA pull-down assay. The miR-324-5p in RNA pulled down by Bio-LINC00491 was determined by RT-PCR. **C** The expression of miR-324-5p in 60 cases of liver cancer and adjacent tissue samples. **D** The effect of LINC00491 over-expression vector (LINC00491) and siLINC00491 on the expression of miR-324-5p in HUH-7 and SK-Hep-1 cell

The potential target miRNAs for LINC00491 were predicted using Starbase. Based on the bioinformatic analysis and preliminary experiments, this study chose miR-324-5p. The 3′-UTR of LINC00491 binding to miR-324-5p was shown in Fig. 4A. An RNA pull-down assay was performed to examine if LINC00491 could bind to miR-324-5p in liver cancer cells. As shown in Fig. 4B, overexpression of LINC00491 led to higher miR-324-5p enrichment as compared to the control, indicating that miR-324-5p could directly bind to LINC00491 (*P* = 0.0059). Next, this research examined the expression of miR-324-5p in the liver cancer tissues and the adjacent non-tumor tissues by qRT-PCR. The results exhibited that miR-324-5p had lower expression in the liver cancer tissues as compared to the normal tissues (*P* < 0.0001) (Fig. 4C). Then, the effect of LINC00491 on miR-324-5p expression was investigated. As shown in Fig. 4D, overexpression of LINC00491 decreased the expression of miR-324-5p (*P* = 0.0070 or *P* = 0.0096), whereas LINC00491 knockdown increased the miR-324-5p expression in HUH-7 and SK-Hep1 cells (*P* = 0.0093 or *P* = 0.0034). This indicated that LINC00491 could directly suppress the expression of miR-324-5p.

### LINC00491 promoted ROCK1 expression by reducing miR-324-5p

**Fig. 5:**
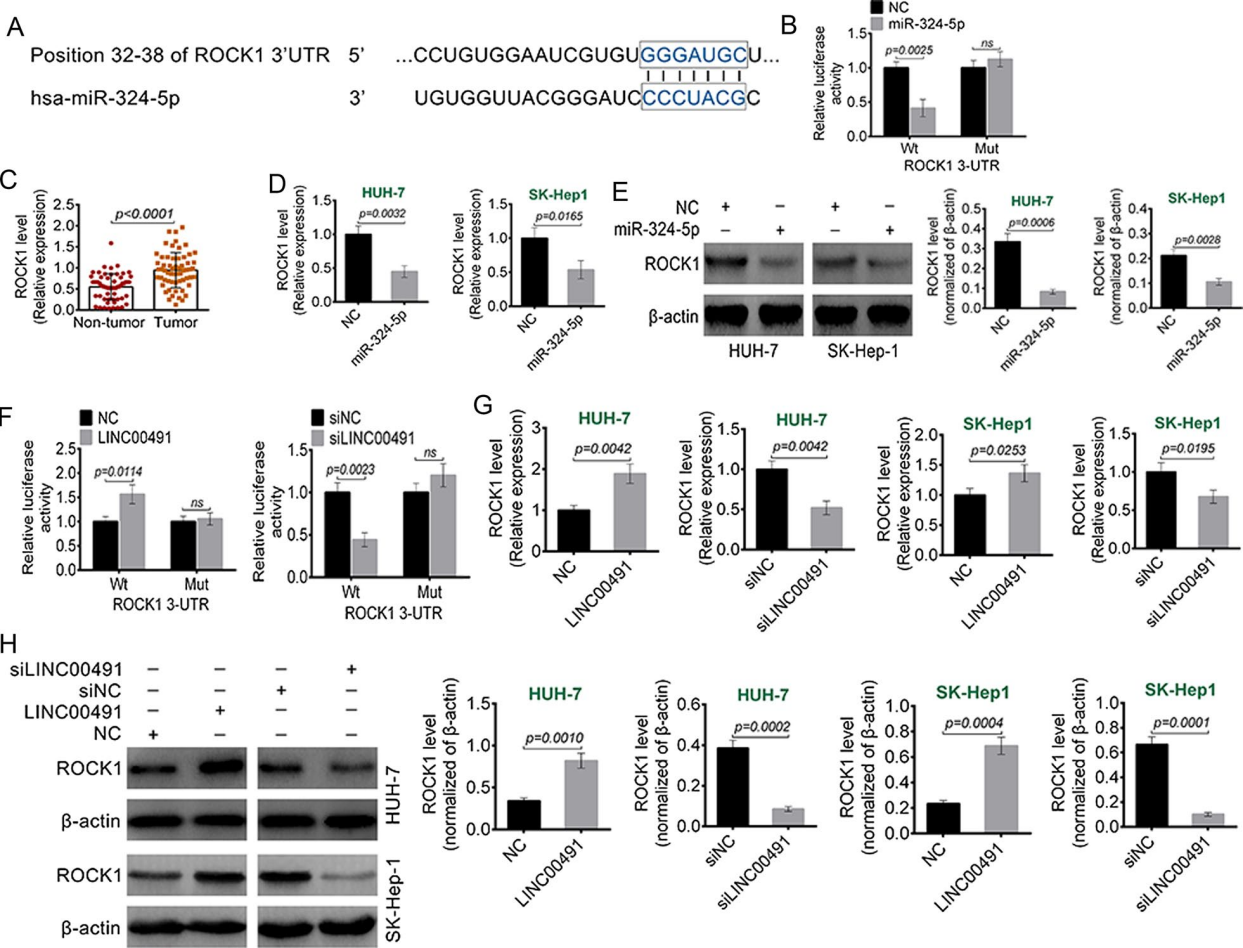
LINC00491 promoted ROCK1 expression by reducing miR-324-5p. **A** The targeting regions of miR-324-5p for ROCK1. **B** Dual luciferase reporter gene assay was performed using HEK-293T cells. Relative luciferase activity in HEK-293T cells co-transfected with ROCK1-WT or ROCK1-MUT luciferase reporter and miR-324-5p NC or mimics. **C** The expression of ROCK1 in 60 cases of liver cancer and adjacent tissue samples. **D**, **E** The effect of miR-324-5p on the mRNA (**D**) and protein (**E**) expression of ROCK1 in HUH-7 and SK-Hep-1 cell. **F** Dual luciferase reporter gene assay was performed using HEK-293T cells. Relative luciferase activity in HEK-293T cells co-transfected with ROCK1-WT or ROCK1-MUT luciferase reporter and LINC00491 over-expression vector (LINC00491) or siLINC00491. **G**, **H** The effect of LINC00491 over-expression vector (LINC00491) and siLINC00491 on the mRNA (**G**) and protein (**H**) expression of ROCK1 in HUH-7 and SK-Hep-1 cell. ns: the difference was not statistically significant

This article predicted the miR-324-5p target by TargetScan. According to the prediction, miR-324-5p could bind to the 3’UTR of ROCK1 (Fig. 5A). Dual-luciferase reporter assay was shown in Fig. 5B. As a result, miR-324-5p mimics could reduce the luciferase activity of the luciferase reporter carrying the wild-type (Wt) sequence of ROCK1-3’UTR (*P* = 0.0025), but not the mutant (Mut) type. qRT-PCR presented that, ROCK1 was highly expressed in the tumor tissues of liver cancer cases than that in normal tissues (*P* < 0.0001) (Fig. 5C). The regulation of miR-324-5p on ROCK1 was investigated by qRT-PCR and Western blotting. As shown in Fig. 5D, E, up-regulation of miR-324-5p significantly reduced mRNA (*P* = 0.0032 or *P* = 0.0165) and protein (*P* = 0.0006 or *P* = 0.0028) expression of ROCK1 in HUH-7 and SK-Hep1 cells. As shown in Fig. 4F, over-expression of LINC00491 increased the luciferase activity of the luciferase reporter carrying the wild-type (Wt) sequence of ROCK1-3’UTR (*P* = 0.0114). Conversely, the luciferase activity of ROCK1 wild-type (Wt) reporter was reduced by LINC00491 knockdown (*P* = 0.0023). As shown in Fig. 5G, H, over-expression of LINC00491 increased ROCK1 protein expression in HUH-7 and SK-Hep1 cells (*P* = 0.0010 or *P* = 0.0004). On the contrary, knockdown of LINC00491 reduced ROCK1 protein expression in HUH-7 and SK-Hep1 cells (*P* = 0.0002 or *P* = 0.0001). This suggested that LINC00491 could promote ROCK1 expression through reducing miR-324-5p.

### miR-324-5p reversed the promotion of LINC00491 on liver cancer cells malignant phenotype

**Fig. 6:**
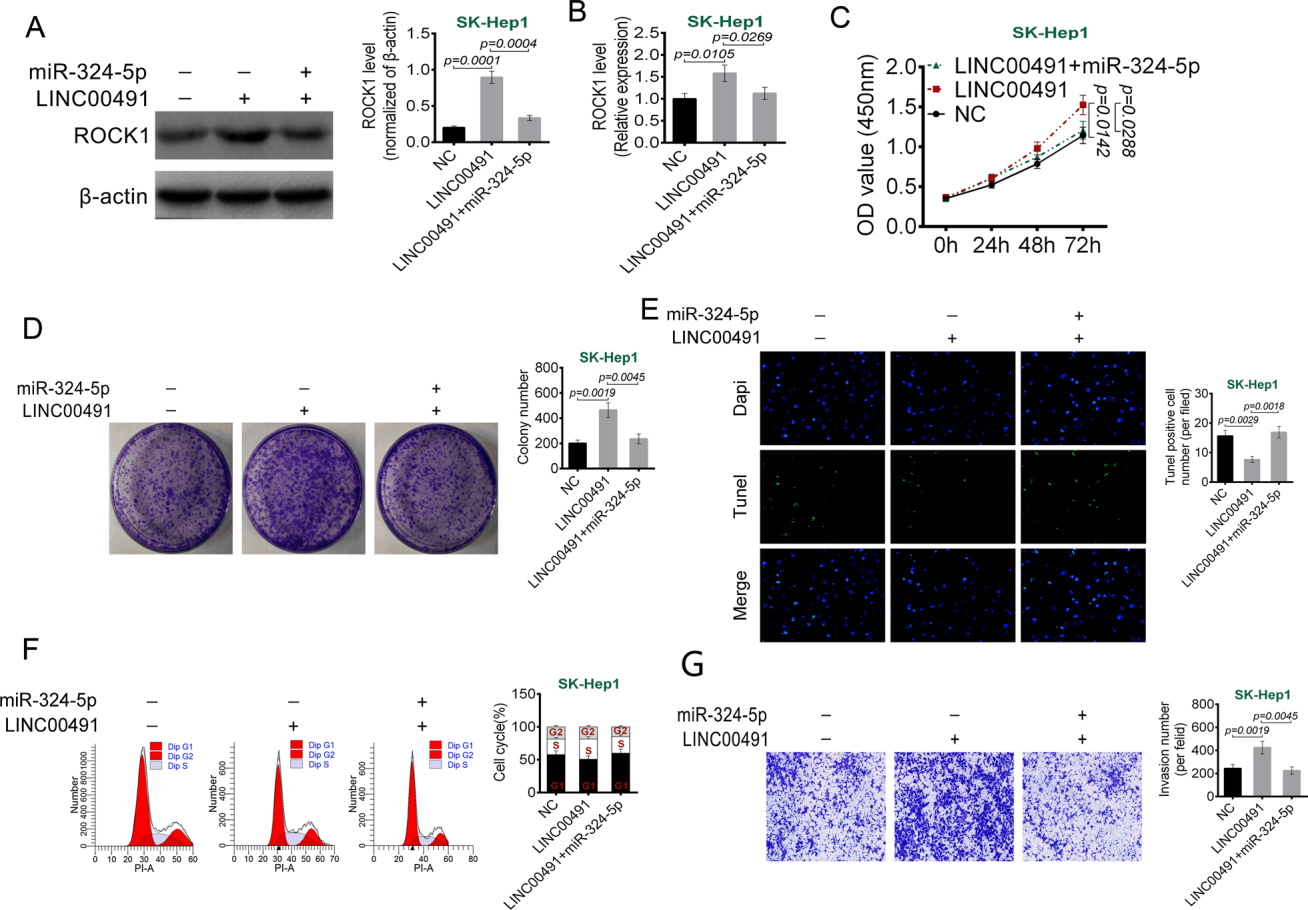
miR-324-5p reversed the promotion of LINC00491 overexpression on liver cancer cells malignant phenotype. **A**, **B** The effect of LINC00491 and miR-324-5p on the protein (**A**) and mRNA (**B**) expression of ROCK1 in SK-Hep-1 cell. **C** The effect of LINC00491 and miR-324-5p on the proliferation of SK-Hep-1 cells as determined by CCK-8 assay. **D**–**G** The effects of LINC00491 and miR-324-5p on the colony formation (**D**), apoptosis (**E**), cell cycle (**F**) and invasion (**G**) in SK-Hep-1 cell

As shown in Fig. 6A, B, relative to NC group, overexpression of LINC00491 significantly promoted the protein (*P* = 0.0001) and mRNA (*P* = 0.0105) expression of ROCK1. However, matched with LINC00491 group, the ROCK1 protein (*P* = 0.0004) and mRNA (*P* = 0.0269) expression was markedly reduced in SK-Hep1 cells of LINC00491 + miR-324-5p group. CCK-8 and colony formation assays exhibited that overexpression of LINC00491 significantly promoted the proliferation (*P* = 0.0142) and colony formation (*P* = 0.0019) of SK-Hep1 cells. However, this effects were abrogated by miR-324-5p mimics (*P* = 0.0288 or *P* = 0.0045) (Fig. 6C–D). Additionally, LINC00491 overexpression prominently reduced apoptosis (*P* = 0.0029) and cells in G1 phase and increased invasion SK-Hep1 cells (*P* = 0.0019). However, miR-324-5p mimics abrogated the above effects (*P* = 0.0018 or *P* = 0.0045) (Fig. 6E–G). These results suggested that miR-324-5p reversed the promotion of LINC00491 on liver cancer cells malignant phenotype.

### ROCK1 knockdown reversed the promotion of LINC00491 on liver cancer cells malignant phenotype

**Fig. 7:**
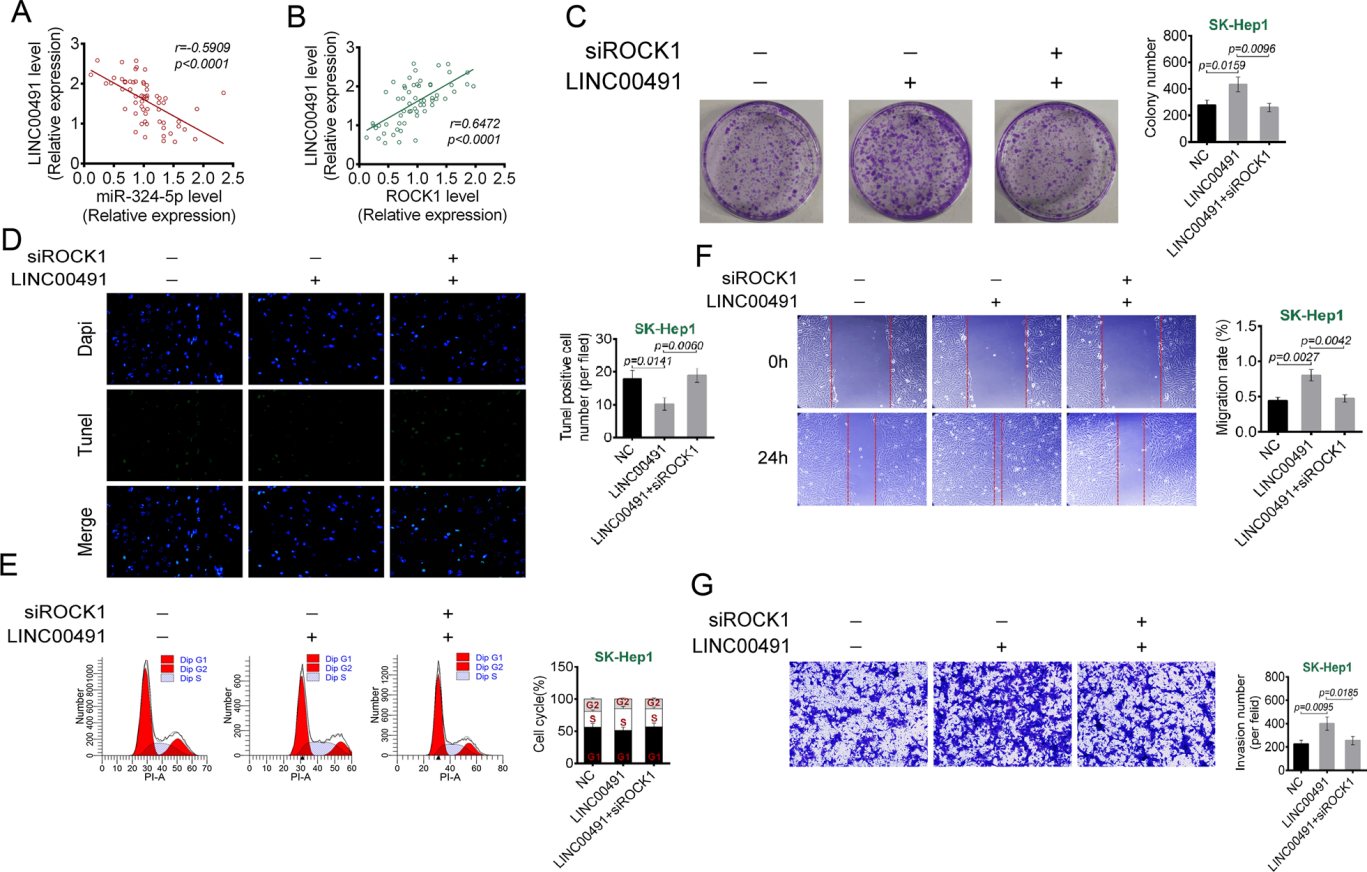
ROCK1 knockdown reversed the promotion of LINC00491 overexpression on liver cancer cells malignant phenotype. **A** The expression of LINC00491 and miR-324-5p in tumor tissues of liver cancer cases was negatively correlated. **B** The expression of LINC00491 and miR-324-5p in tumor tissues of liver cancer cases was positively correlated. **C**–**G** The effect of LINC00491 and siROCK1 on the colony formation (**C**), apoptosis (**D**), cell cycle (**E**), migration (**F**) and invasion (**G**) in SK-Hep-1 cell

As shown in Fig. 7A, B, there was a negative correlation between LINC00491 and miR-324-5p expression (r = − 0.5909, *P* < 0.0001) and a positive correlation between LINC00491 and ROCK1 expression (r = 0.6472, *P* < 0.0001). As shown in Fig. 7C, overexpression of LINC00491 promoted colony formation of SH-Hep1 cells (*P* = 0.0159), whereas siROCK1 abrogated this effect (*P* = 0.0096). Consistently, LINC00491 overexpression declined apoptosis (*P* = 0.0141), cells in G1 phase, and elevated migration (*P* = 0.0027) and invasion (*P* = 0.0095) SK-Hep1 cells. Intriguingly, siROCK1 abrogated the above effect (*P* = 0.0060, *P* = 0.0042 or *P* = 0.0185) (Fig. 7D–G). Taken together, ROCK1 knockdown reversed the promotion of LINC00491 on liver cancer cells malignant phenotype.

### LINC00491 promoted tumor growth and metastasis in vivo

**Fig. 8:**
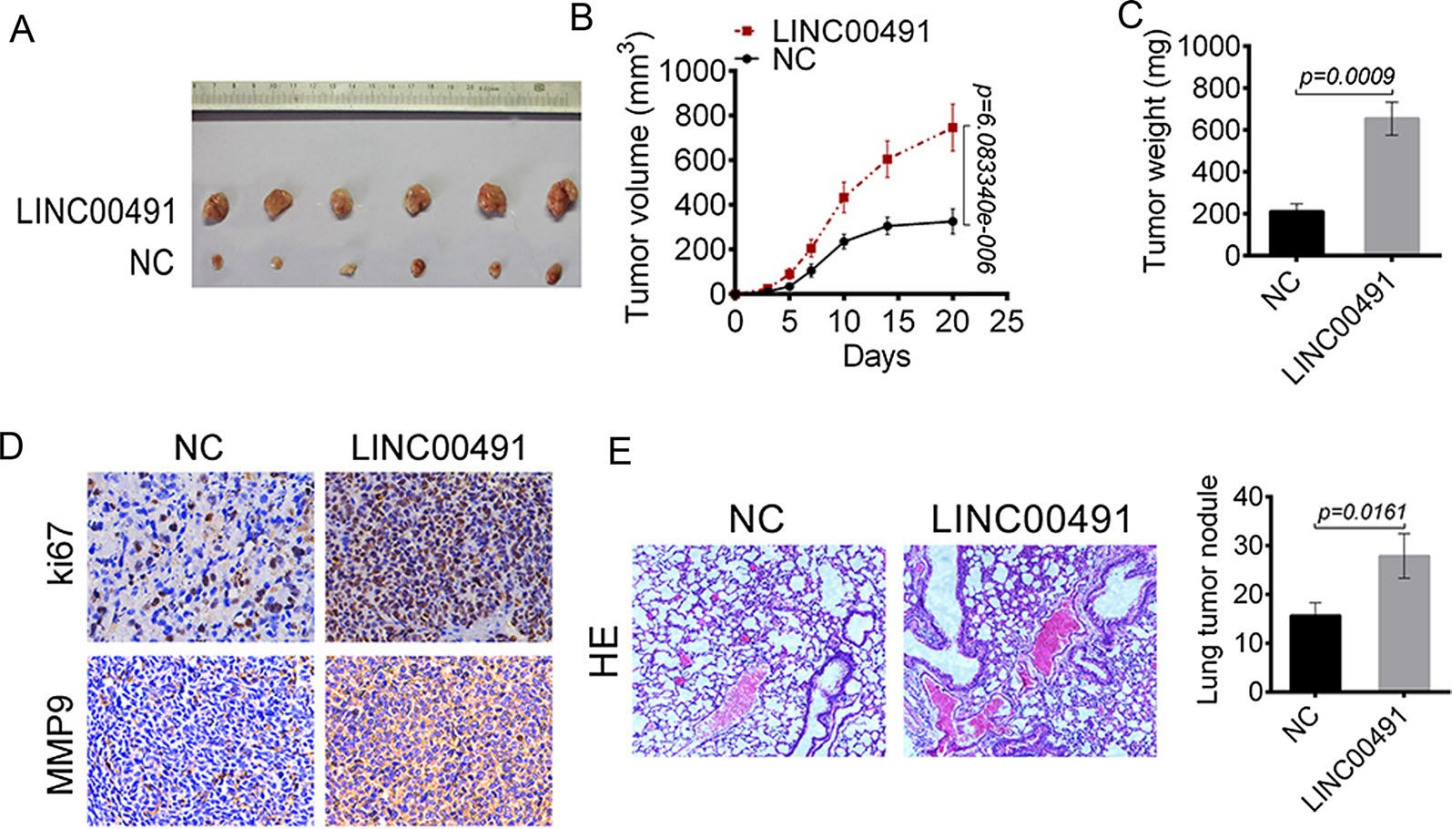
LINC00491 promoted tumor growth and metastasis in vivo. **A** The images of xenograft tumors in mice injected with SK-Hep-1 cells transfected by LINC00491 over-expression vectors and NC. **B** The tumor volume was measured every 3 days. **C** The weight of the tumor tissues was measured immediately after resected. **D** The Ki-67 and MMP9 positive cells in xenograft tumors of nude mice. **E** The hematoxylin–eosin staining of lung tissues of mice injected with SK-Hep-1 cells transfected by LINC00491 over-expression vectors and NC. The number of lung tumor nodule was shown in the right panel

The in vivo role of LINC00491 in tumor growth and lung metastasis was evaluated. As shown in Fig. 8A–C, the tumor volume and weight from the LINC00491 overexpression group was significantly higher as compared to the control group (*P* < 0.01). Ki67 can reflect the proliferative activity of tumor cells and MMP-9 is an indispensable factor for tumor invasion and metastasis. The expressions of Ki67 and MMP-9 in the tumor tissues were detected by IHC staining. As shown in Fig. 8D, a large number of Ki67-positive and MMP-9-positive cells were present in the LINC00491 overexpression group. As shown in Fig. 8E, indeed, a large number of lung tumor nodules were observed in the LINC00491 overexpression group as relative to NC group (*P* = 0.0161). These data indicated that LINC00491 overexpression enhanced liver cancer cells growth and lung metastasis in vivo.

## Discussion

LncRNAs consisting of more than 200 nucleotides are a class of non-coding RNAs. It is originally thought as a by-product of RNA polymerase II transcription contributing to "noise" during transcription [20]. Recent study shows that lncRNAs exhibits potentially complex and diverse biological functions, such as regulation of tumor suppressor/anticancer gene expression and signaling transduction [21]. LINC00491 is a recently discovered tumor-associated lncRNA. Several studies show that LINC00491 expression is positively correlated with the overall survival in various malignancies such as breast cancer [22] and colorectal cancer [18]. It can be used as an independent prognostic biomarker for endometrial cancer [19]. However, the biological functions underlying tumor growth, metastasis and invasion have not yet been reported.

This study initially investigated the biological function of LINC00491 in liver cancer. TCGA and GEO databases analysis showed that the expression of LINC00491 in liver cancer tissues was significantly higher as compared to the adjacent tissues. Then the expression of LINC00491 in the clinical specimens of 60 liver cancer cases was researched by RT-PCR. Similar results were found. The analysis of the relationship between LINC00491 expression and the clinicopathological characteristics of liver patients suggested that high LINC00491 expression was closely correlated with poor tumor TNM stage and metastasis. In addition, the expression of LINC00491 in various liver cancer cell lines was significantly higher as compared to the normal liver cells. These results suggested that LINC00491 might play an important role in the pathogenesis of liver cancer. It might be a potential therapeutic target for liver cancer. To evaluate the potential role of LINC00491 in liver cancer progression, this article investigated the effects of over-expression and knockdown of LINC00491 in cell biological behaviors. The results suggested that LINC00491 over-expression exhibited pro-tumor effects by promoting proliferation, cell cycle progression, migration and invasion, and inhibition of apoptosis. Conversely, LINC00491 knockdown showed anti-tumor activity. Thus, LINC00491 might be a novel target for the treatment of liver cancer.

LncRNAs can regulate the transcription efficiency and splicing form by its interaction with the promoter region or the formation of a complementary double strand with the transcript of its target genes [23]. In tumors, lncRNAs usually affects the tumorigenesis, progression and biological behaviors by regulating the expression of miRNAs [9]. This study showed that the 3' UTR of LINC00491 contained binding sites for miR-324-5p. The biological effects of miR-324-5p in some cancers have been reported in recent years. For instance, Jiang et al. showed that miR-324-5p was a target miRNA of lncRNA tumor protein translationally controlled 1 antisense RNA 1 (TPT1-AS1). It could inhibit proliferation, colony formation, migration, invasion and epithelial-mesenchymal transition (EMT) in cervical cancer cells [24]. Lin et al. researched that miR-324-5p was significantly higher expressed in lung cancer cells. It increased the proliferation of lung cancer cells [25]. On the opposite, Cao et al. explored that miR-324-5p was down-regulated in hepatocellular carcinoma tissues and cells [26]. This study indicated that miR-324-5p had lower expression in liver cancer tissues and cells.

At present, there are three main widely accepted mechanisms for lncRNAs regulation of miRNAs, as follows: (1) the lncRNAs can compete with miRNAs for the 3'-UTR of target gene mRNAs, thus indirectly inhibiting the negative regulation of miRNAs on the target genes; (2) some lncRNAs can form pre-miRNAs by intracellular cleavage, and then produce specific miRNAs to regulate the expression of target genes; (3) some lncRNAs can function as endogenous miRNAs sponges, thereby inhibiting miRNAs expression and indirectly affecting the malignant biological behavior of tumor cells [27]. This article indicated that LINC00491 could negatively regulate the expression of miR-324-5p in liver cancer cells. However, whether LINC00491 inhibits the expression of miR-324-5p by competitively binding to the 3'-UTR of miR-324-5p or exerts its function as an endogenous miRNA sponge still needs to be verified in the future.

Study has shown that miRNAs regulates the post-translational expression of proteins by binding to the 3'-UTR of its target gene mRNAs, thereby regulating the biological function of cells [28]. This study revealed that miR-324-5p had a complementary binding sequence for ROCK1. ROCK1 is a serine/threonine-protein kinase involved in the regulation of cell division, proliferation and skeletal formation by regulating the downstream target effector molecules [29]. Recent studies show that abnormal expression of ROCK1 plays an important role in the occurrence and development of various tumors, such as breast cancer, prostate cancer and gastric cancer [30, 31]. In this research, it was shown that ROCK1 was highly expressed in liver cancer tissues. miR-324-5p negatively regulated the expression of ROCK1 and LINC00491 positively regulated the expression of ROCK1. In addition, miR-324-5p not only counteracted the regulation of LINC00491 on ROCK1, but also counteracted the effects of LINC00491 on the proliferation, apoptosis, migration and invasion of liver cancer cells. Moreover, ROCK1 knockdown counteracted the effects of LINC00491 on the biological behaviors of liver cancer cells. Taken together, the promotion effects of LINC00491 on liver cancer progression might be through enhancing ROCK1 expression via directly reducing miR-324-5p.

Additionally, in vivo experiment was performed. The results indicated that LINC00491 overexpression enhanced liver cancer cells growth and metastasized to the lungs in mice. Thus, LINC00491 overexpression facilitated liver cancer progression in vivo. It was better to research the effect of LINC00491 knockdown on liver cancer progression in vivo. However, due to the limitations of the laboratory, we are currently unable to carry out this experiment. This point will be the focus of our future research.

In conclusion, this study suggested that LINC00491 was up-regulated in liver cancer tissues and cells. High LINC00491 expression was closely related to poor TNM stage and metastasis. LINC00491 showed a tumor-promoting effect in vitro, while si-LINC00491 showed anti-tumor activity. miR-324-5p or si-ROCK1 counteracted the promotion of LINC00491 on liver cancer cells malignant phenotype. Overall, this study indicated that LINC00491 might play a promotion role in the development and metastasis of liver cancer through miR-324-5p/ROCK1. LINC00491 might be a potential treatment target for liver cancer.

## Data Availability

The data of this study is availability on a reasonable request.

## Abbreviations

HBV: Hepatitis B virus
HCV: Hepatitis C virus
FISH: Fluorescence in situ hybridization
IHC: HE and immunohistochemistry
TCGA: Cancer Genome Atlas
GEO: Gene Expression Omnibus
Wt: Wild-type
Mut: Mutant
